# The Complete Chloroplast Genome Sequence of Tree of Heaven (*Ailanthus altissima* (Mill.) (Sapindales: Simaroubaceae), an Important Pantropical Tree

**DOI:** 10.3390/ijms19040929

**Published:** 2018-03-21

**Authors:** Josphat K. Saina, Zhi-Zhong Li, Andrew W. Gichira, Yi-Ying Liao

**Affiliations:** 1 Fairy Lake Botanical Garden, Shenzhen & Chinese Academy of Sciences, Shenzhen 518004, China; jksaina@wbgcas.cn; 2 Key Laboratory of Aquatic Botany and Watershed Ecology, Wuhan Botanical Garden, Chinese Academy of Sciences, Wuhan 430074, China; wbg_georgelee@163.com (Z.-Z.L.); gichira@wbgcas.cn (A.W.G.); 3 University of Chinese Academy of Sciences, Beijing 100049, China; 4 Sino-African Joint Research Center, Chinese Academy of Sciences, Wuhan 430074, China

**Keywords:** *Ailanthus altissima*, chloroplast genome, microsatellites, Simaroubaceae, Sapindales

## Abstract

*Ailanthus altissima* (Mill.) Swingle (Simaroubaceae) is a deciduous tree widely distributed throughout temperate regions in China, hence suitable for genetic diversity and evolutionary studies. Previous studies in *A. altissima* have mainly focused on its biological activities, genetic diversity and genetic structure. However, until now there is no published report regarding genome of this plant species or Simaroubaceae family. Therefore, in this paper, we first characterized *A. altissima* complete chloroplast genome sequence. The tree of heaven chloroplast genome was found to be a circular molecule 160,815 base pairs (bp) in size and possess a quadripartite structure. The *A. altissima* chloroplast genome contains 113 unique genes of which 79 and 30 are protein coding and transfer RNA (tRNA) genes respectively and also 4 ribosomal RNA genes (rRNA) with overall GC content of 37.6%. Microsatellite marker detection identified A/T mononucleotides as majority SSRs in all the seven analyzed genomes. Repeat analyses of seven Sapindales revealed a total of 49 repeats in *A. altissima*, *Rhus chinensis*, *Dodonaea viscosa*, *Leitneria floridana*, while *Azadirachta indica*, *Boswellia sacra*, and *Citrus aurantiifolia* had a total of 48 repeats. The phylogenetic analysis using protein coding genes revealed that *A. altissima* is a sister to *Leitneria floridana* and also suggested that Simaroubaceae is a sister to Rutaceae family. The genome information reported here could be further applied for evolution and invasion, population genetics, and molecular studies in this plant species and family.

## 1. Introduction

*Ailanthus altissima* (Mill.) Swingle, a deciduous tree in the Simaroubaceae family, is widely distributed throughout temperate regions in China. It grows rapidly reaching heights of 15 m (49ft) in 25 years and can tolerate various levels of extreme environments (e.g., low temperatures, sterile soils, arid land). Besides, it reproduces through sexual (seeds disperse by wind) or asexual (sprouts) methods [1]. Two hundred years ago it was brought to Europe and North America. *A. altissima* being an early colonizer can survive high levels of natural or human disturbance [2]. Therefore, in recent years, it is commonly known as an exotic invasive tree developed into an invasive species expanding on all continents except Antarctica [1]. While previous studies in *A. altissima* have mainly focused on discovering the biological features of this plant to prevent its expansion, there is limited information to understand the impact of genetic diversity and evolution. Thus, genomic information of *A. altissima* is essential for further molecular studies, identification, and evolutionary studies. 

Many studies have analyzed the genetic diversity of *A. altissima* using various markers, for example, chloroplast DNA [2,3], microsatellite primer [4,5]. These studies provided a detailed series of information about genetic structure and genetic diversity of *A. altissima* in native and invasive area. However, to understand the genetic diversity and population structure within *A. altissima* natural populations, more genetic resources are required. 

It is well known that chloroplasts (cp) are key organelles in plants, with crucial functions in the photosynthesis and biosynthesis [6]. Current research shows that chloroplast genomes in angiosperms have highly conserved structure, gene content, organization, compared with either nuclear or mitochondrial genomes [7,8]. In general, cp genomes in angiosperms have circular structure consisting of two inverted repeat regions (IRa and IRb) that divides a large–single-copy (LSC) and a small-single-copy (SSC) regions [9]. Nevertheless, mutations, duplications, arrangements and gene loss have been observed, including the loss of the inverted repeat region in leguminous plants [7,10,11,12]. Some studies have applied plant plastomes to study population genetic analyses and basal phylogenetic relationships at different taxonomic levels [13], also to investigate the functional and structural evolution in plants [14,15,16]. At present, more cp genomes have been sequenced as a result of next-generation sequencing technologies advancement resulting in low sequencing costs. 

More than 800 sequenced plastomes from various land plants have boosted our understanding of intracellular gene transfer, conservation, diversity, and genetic basis [17]. Although cp genomes have been sequenced in many trees such as *Castanea mollissima* [18]), *Liriodendron tulipifera* [19], *Eucalyptus globules* [20], and *Larix deciduas* [21], the plastome of *Leitneria floridana* (GenBank NC_030482) a member of Simaroubaceae has been sequenced but no analysis has been published at present despite the family containing many high economic value trees. Regardless of its potential use in crop or tree species improvement, studies on invasive species such as *A. altissima* which is also an important economic tree in the North China are too few. Chloroplast genome sequencing in invasive species could bring insights into evolutionary aspects in stress-tolerance related trait and genetic variation.

Simple sequence repeat (SSR) also called microsatellite markers are known to be more informative and versatile DNA-based markers used in plant genetic research [22]. These DNA markers are reliable molecular tools that can be used to examine plants genetic variation. SSR loci are evenly distributed and very abundant in angiosperms plastomes [23,24]. Chloroplast microsatellites are typically mononucleotide tandem repeats, and SSR in the fully sequenced genome could be used in plant species identification and diversity analysis. CpSSR in the fully sequenced plants plastomes such as; orchid genus *Chiloglottis* [25], *Cryptomeria japonica* [26], *Podocarpus lambertii* [27], *Actinidia chinensis* [28], have proven to be useful genetic tools in determining gene flow and population genetics of cp genomes. However, the lack of published plastome of *A. altissima* has limited the development of suitable SSR markers.

Here, we sequenced the complete chloroplast genome of *A. altissima*, and characterized its organization and structure. Furthermore, phylogenetic relationship using protein coding genes from selected species, consisting of 31 species from five families was uncovered for the Simaroubaceae family within the order Sapindales. Lastly, this resource will be used to develop SSR markers for analyzing genetic diversity and structure of several wild populations of *A. altissima*.

## 2. Results and Discussion

### 2.1. Ailanthus altissima Genome Size and Features

**Figure 1:**
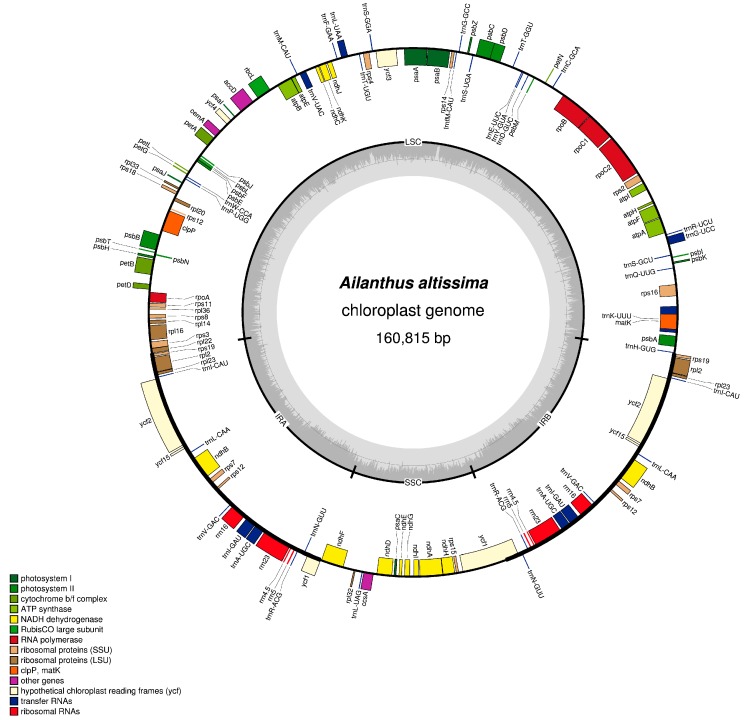
Circular gene map of *A. altissima* complete chloroplast genome. Genes drawn on the outside of the circle are transcribed clockwise, whereas those inside are transcribed clockwise. The light gray in the inner circle corresponds to AT content, while the darker gray corresponds to the GC content. Large single copy (LSC), Inverted repeats (IRa and IRb), and Small single copy (SSC) are indicated.

The *A. altissima* chloroplast genome has a quadripartite organizational structure with overall size of 160,815 base pairs (bp) including two copies of Inverted repeats (IRa and IRb) (27,051 bp each) separating the Large Single Copy (LSC) (88,382 bp) and Small Single Copy Region (SSC) (18,332 bp) (Figure 1). Notably, the genome content; gene order, orientation and organization of *A. altissima* is similar to the reference genome and other sequenced Sapindales plastomes [29,30] with genome size of about 160 kb. The overall guanine-cytosine (GC) content of the whole genome is 37.6%, while the average adenine-thymine A + T content is 62.36%. The relatively higher IR GC content and A + T bias in this chloroplast have been previously reported in genomes of relative species in order Sapindales [31]. The GC content of the LSC, SSC and IR regions are 35.7, 32.2 and 42.6% respectively. Moreover, the protein coding sequences had 38.3% GC content.

The tree of heaven (*A. altissima*) chloroplast genome harbored a total of 113 different genes, comprising 79 protein coding genes (PCGS), 30 transfer RNA (tRNA) genes, and four ribosomal RNA (rRNA) genes (Table 1). All the 77 PCGS started with the common initiation codon (ATG), but *rps19* and *ndhD* genes started with alternative codons GTG and ACG respectively, this unusual initiator codons have been observed to be common in other angiosperm chloroplast genomes [32,33,34]. Of the 79 protein coding sequences, 60 are located in the LSC, 11 in the SSC and eight genes were duplicated the IR region, while 22 tRNA genes were found in LSC, seven replicated in the IR region and one located in the SSC region.

Similar to some closely related plant species in order Sapindales, the chloroplast genome of *A. altissima* has maintained intron content [35]. Among the 113 unique genes, the *rps16*, *rpoC1*, *petB*, *rpl2*, *ycf15*, *ndhB*, *ndhA*, *atpF*, six tRNA genes (trnG-GCC*, trnA-UGC*, trnL-UAA*, trnI-GAU*, trnK-UUU*, trnV-UAC*) possess one intron, and *ycf3* and *clpP* genes harbored two introns. The *rps12* trans-splicing gene has two 3′ end exons repeated in the IRs and the 5′ end exon located in the LSC region, which is similar to that in *C. platymamma* [30], *C. aurantiifolia* [29], *Dipteronia* species [35]. The *ycf1* gene crossed the IR/SSC junction forming a pseudogene *ycf1* on the corresponding IR region. The *rps19* gene in *A. altissima* was completely duplicated in the inverted repeat (IR) region, which most other chloroplast genomes have presented [29,30].

### 2.2. IR Expansion and Contraction and Genome Rearrangement 

**Figure 2:**
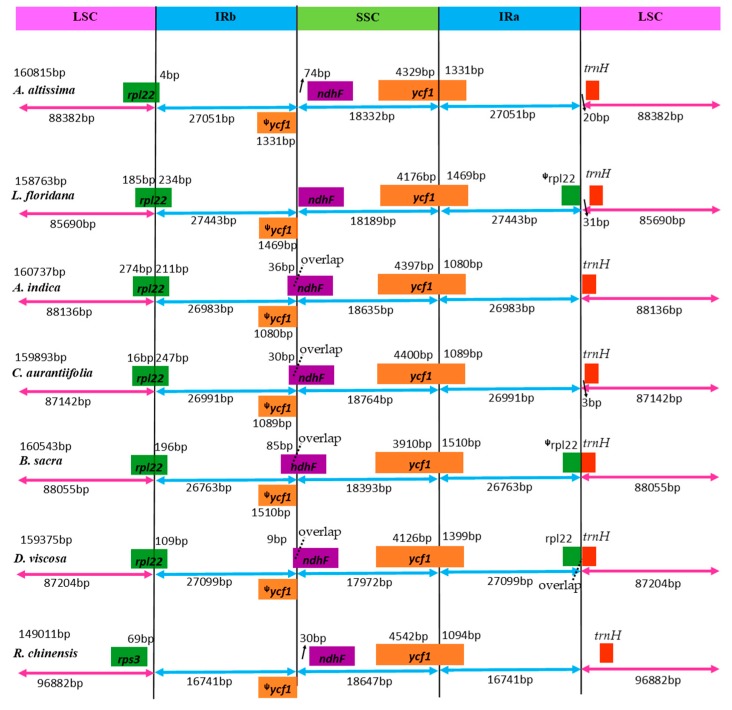
Comparison of IR, LSC and SSC junction positions among seven Chloroplast genomes. The features drawn are not to scale. The symbol ᵠ means pseudogene created by IRb/SSC border extension into *ycf1* genes. Colored boxes for genes represent the gene position.

The angiosperms chloroplast genomes are highly conserved, but slightly vary as a result of either expansion or contraction of the single-copy (SC) and IR boundary regions [36]. The expansion and contraction of the IR causes size variations and rearrangements in the SSC/IRa/IRb/LSC junctions [37]. Therefore, in this study, exact IR boundary positions and their adjacent genes of seven representative species from different families in order Sapindales were compared (Figure 2). The functional *ycf1* gene crossed the IRa/SSC boundary creating *ycf1* pseudogene fragment at the IRb region in all the genomes. Besides, *ycf1* pseudogene overlapped with the *ndhF* gene in the SSC and IRa junctions in four genomes with a stretch of 9 to 85 bp, but *ndhF* gene is located in SSC region in *L. floridana*, *R. chinensis* and *A. altissima*.

The *rpl22* gene crossed the LSC/IRb junction in all the chloroplast genomes except in *R. chinensis*. Furthermore, this gene was partially duplicated forming a pseudogene fragment at the corresponding IRA/LSC junction in *L. floridana* and *B. sacra*, but completely duplicated in *D. viscosa*. In all the seven chloroplast genomes, the *trnH-GUG* gene was located in the LSC regions, however this gene overlapped with *rpl22* gene in *D. viscosa*. The results reported here are congruent with the recent studies which showed that the *trnH-GUG* gene was situated in the LSC region in some species from order Sapindales, while the SSC/IRa border extends into the protein coding gene *ycf1* with subsequent formation of a *ycf1* pseudogene [29,30]. Despite the seven chloroplast genomes of Sapindales having well-conserved genomic structure in terms of gene order and number, length variation of the whole chloroplast genome sequences and LSC, SSC and IR regions was detected among these genomes. This sequence variation might have been as a result of boundary expansion and contraction between the single copy and IR boundary regions among plant lineages as suggested by Wang and Messing 2011 [38].

**Figure 3:**
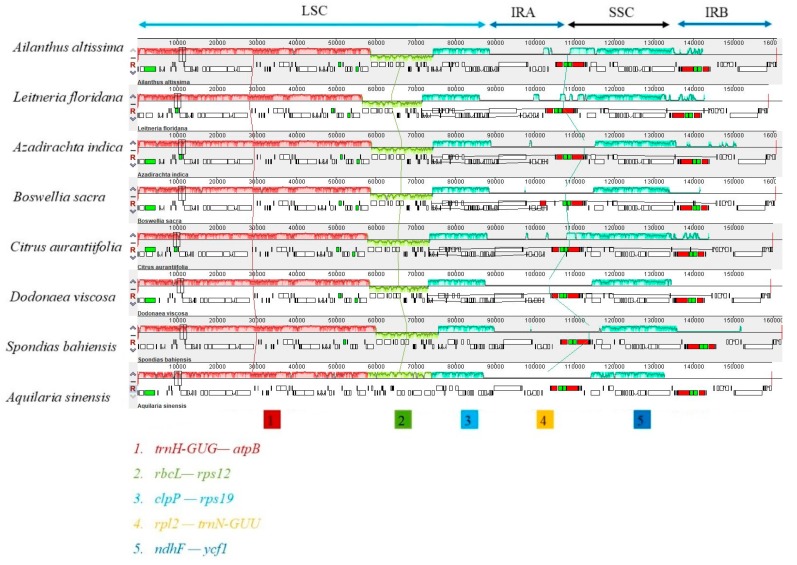
Gene arrangement map of seven chloroplast genomes representing families from Sapindales, and one reference species (*Aquilaria sinensis*) aligned using Mauve software Local collinear blocks within each alignment are represented in as blocks of similar color connected with lines. Annotations of rRNA, protein coding and tRNA genes are shown in red, white and green boxes respectively.

The mauve alignment for seven species revealed that all the genomes formed collinear blocks (LCBs). In particular, all the seven species; *A. altissima*, *Leitneria floridana*, *Azadirachta indica*, *Citrus aurantiifolia*, *Boswellia Sacra*, *Spondias bahiensis* and *Dodonaea viscosa* reveal a syntenic structure, however block two was inverted (from *rpl20* to *rbcL* genes) compared to the reference genome (*Aquilaria sinensis*). The collinear blocks of the genes including ribosomal RNA, tRNA, and protein coding genes revealed that all the seven genomes were relatively conserved with no gene rearrangement (Figure 3). Some other studies have revealed homology in genome organization and no gene rearrangements, thus our findings support their conclusions [31,39,40].

### 2.3. Codon Usage and Putative RNA Editing Sites in Chloroplast Genes of A. altissima

**Figure 4:**
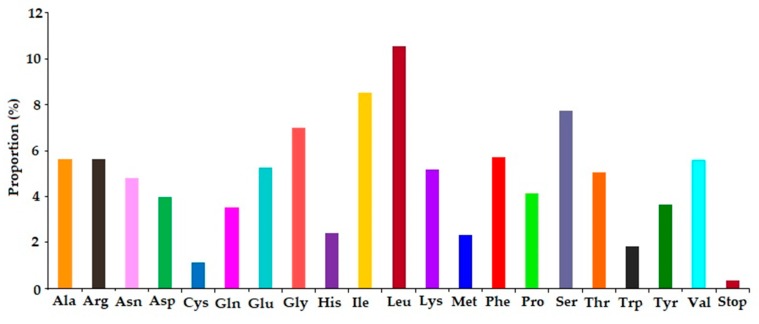
Amino acid frequencies in *A. altissima* chloroplast genome protein coding sequences.

In this study, we analyzed codon usage frequency and the relative synonymous codon usage (RSCU) in the *A. altissima* plastome. All the protein coding genes presented a total of 68,952 bp and 22,964 codons in *A. altissima* chloroplast genome. Of 22,964 codons, leucine (Leu) being the most abundant amino acid had a frequency of 10.56%, then isoleucine (Ile) with 8.54%, while cysteine (Cys) was rare with a proportion of 1.12% (Tables S1 and S2, Figure 4). Our study species genome is like other previously reported genomes which showed that leucine and isoleucine are more common [41,42,43,44,45]. Furthermore, comparable to other angiosperm chloroplast genomes, our results followed the trend of codon preference towards A/T ending which was observed in plastomes of two *Aristolochia* species [46], *Scutellaria baicalensis* [47], *Decaisnea insignis* [34], *Papaver rhoeas* and *Papaver orientale* [48] *Cinnamomum camphora* [49], and *Forsythia suspensa* [41]. All the twenty-eight A/U—ending codons had RSCU values of more than one (RSCU > 1), whereas the C/G—ending codons had RSCU values of less than one. Two amino acids, Methionine (Met) and tryptophan (Trp) showed no codon bias. The results for number of codons (Nc) of each protein coding gene ranged from 38.94 (*rps14* gene) to 58.37 (*clpP* gene).

The potential RNA editing sites in tree of heaven chloroplast genome was done using PREP program which revealed that most conversions at the codon positions change from serine (S) to leucine (L) (Table 2). In addition, 15 (27.78%), 39 (72.22%), and 0 editing locations were used in the first, second and third codons respectively. One RNA editing site converted the amino acid from apolar to polar (proline (P) to serine (S). Overall, the PREP program identified a total of 54 editing sites in 21 protein coding genes, with *ndhB* and *ndhD* genes predicted to have the highest number of editing sites (9). Followed by *ndhA*, *matK*, *rpoC2*, and *rpoB* with four editing sites, whereas *ndhF* had three sites. Interestingly, fifty three of fifty four RNA editing conversions in the *A. altissima* chloroplast genome resulted into hydrophobic products comprising; isoleucine, leucine, tryptophan, tyrosine valine, methionine, and phenylalanine. In general our results are congruent with the preceding reports which also found that most RNA editing sites led to amino acid change from polar to apolar, resulting in increase in protein hydrophobicity [41,46,50]. 

Comparisons of RNA editing sites with other six species from other families revealed that *R. chinensis* and *D. viscosa* have high RNA editing sites (61 each distributed in 20 and 17 genes respectively) followed by *B. sacra* (57 in 20 genes), *A. altissima* (54 in 21 genes), *A. indica* (53 in 21 genes), *C. aurantiifolia* (52 in 21 genes), and *L. floridana* 48 in 20 genes. As shown in Table S3, these results are consistent with several studies in that all the RNA editing sites predicted among the seven species are cytidine (C) to uridine (U) conversions [41,50,51,52]. Majority of RNA editing occurred at the second positions of the codons with a frequency from 62.30% (38/61) in *D. viscosa* to 81.28% (39/48) in *L. floridana*, which concurs with previous plastid genome studies in other land plants [53,54]. All the species shared 19 editing sites distributed in twelve genes (Table 3), whereas the two species from Simaroubaceae family (*L. floridana* and *A. altissima*) shared 33 editing sites in 16 genes this implies that the RNA editing sites in these two species are highly conserved (Table S4). Like previous studies [41,51,55], the *ndhB* gene in most of species analyzed here have the highest number of editing sites. Notably, a RNA editing event was detected at the initiator codon (ACG) resulting in ATG translational start codon in the *ndhD* gene.

### 2.4. Repeat Sequence Analysis

**Figure 5:**
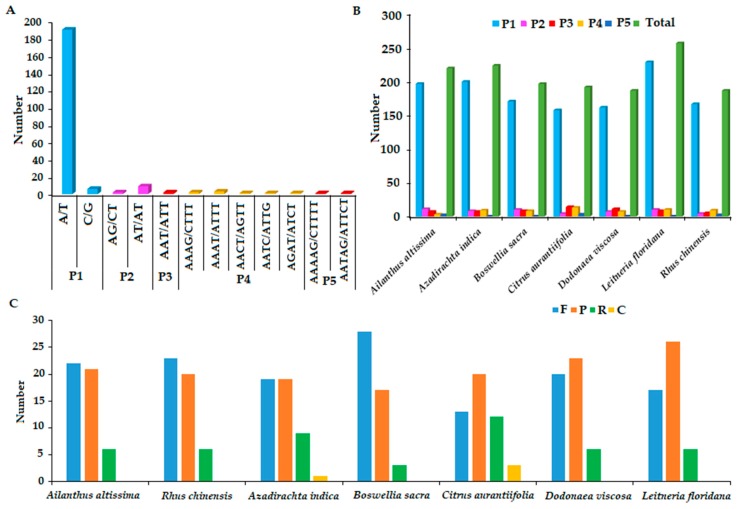
Simple sequence repeat (SSRs) type, distribution and presence in *A. altissima* and other representative species from Sapindales. (**A**) Number of detected SSR motifs in different repeat types in *A. altissima* Chloroplast genome. (**B**) Number of identified repeat sequences in seven chloroplast genomes. (**C**) Number of different SSR types in seven representative species. F, indicate (forward), P (palindromic), R (reverse), and C (complement), while P1, P2, P3, P4, P5 indicates Mono-, di-, tri-, tetra-, and penta-nucleotides respectively. F: forward; P: palindromic, R: reverse; C: complement.

Microsatellites are usually 1–6 bp tandem repeat DNA sequences and are distributed throughout the genome. The presence of microsatellites was detected in the chloroplast genome of *A. altissima* (Figure 5). A total of 219 simple sequence repeats (SSRs) loci were detected, of which mononucleotide repeats occurred with high frequency constituting 190 (86.76%) of all the SSRs. Majority of mononucleotides composed of poly A (polyadenine) (39.27%) and poly T (polythymine) (47.49%) repeats, whereas poly G (polyguanine)or polyC (polycytosine) repeats were rather rare (2.74%). Among the dinucleotide repeat motifs AT/AT were more abundant, while AG/CT were less frequent. One trinucleotide motif (AAT/ATT), five tetra-(AAAG/CTTT, AAAT/ATTT, AACT/AGTT, AATC/ATTG and AGAT/ATCT) and two pentanucleotide repeats (AAAAG/CTTTT and AATAG/ATTCT) were identified. Hexanucleotide repeats were not detected in the *A. altissima* chloroplast genome. 

As shown in Figure 5, the SSR analysis for seven species showed that *Leitneria floridana* had the highest number of SSRs (256) while *Dodonaea viscosa* and *Rhus chinensis* had the lowest (186). In all the seven species, mononucleotide repeats were more abundant with A/T repeats being the most common repeats. This result is consistent with earlier studies in [31,35,46] which revealed that many angiosperm chloroplast genomes are rich in poly A and poly T. Moreover, in the seven analyzed species, hexanucleotide repeats were not detected, whereas *Azadirachta indica*, *Dodonaea viscosa* and *Leitneria floridana* had no pentanucleotide repeats. 

The REPuter program revealed that *A. altissima* chloroplast genome contains 21 palindromic (p), 22 forward (f) and six reverse (r) repeats, however the complement repeats were not detected (Table 4). We notice that all the identified tandem repeats in *A. altissima* were more than 20 bp, while thirteen had length of more than 30 bp. Repeat analyses of seven Sapindales revealed a total of 48 or 49 repeats for each species, with all species containing forward, palindromic and reverse repeats (Figure 5). Compliment repeats were not identified in other species except for *Azadirachta indica* and *Citrus aurantiifolia* which had one and three repeats respectively. *Citrus aurantiifolia* had the highest number of reverse repeats but also lowest number of forward repeats. Most of the repeat lengths were less than 50 bp, however *Boswellia sacra* chloroplast had seven forward repeats with length of between 65 to 251 bp. Besides, we found out that almost all the repeat sequences were located in either IR or LSC region.

### 2.5. Phylogenetic Tree

**Figure 6:**
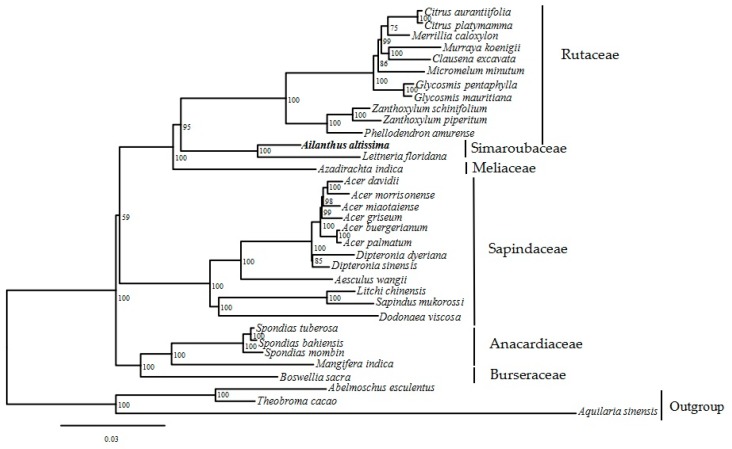
Phylogenetic tree of 31 Sapindales species with three outgroup Malvales species inferred from ML (Maximum likelihood) based on common protein coding genes. The position of *A. altissima* is shown in bold, while bootstrap support values are shown at each node.

The phylogenetic position of *A. altissima* within Sapindales was carried out using 75 protein coding sequences shared by thirty-one taxa from Sapindales (Table S5). Three remaining species were from Thymelaeaceae family (*Aquilaria sinensis*) and Malvaceae (*Theobroma cacao* and *Abelmoschus esculentus*) from order Malvales selected as outgroups (Figure 6). The maximum likelihood (ML) analysis produced a phylogenetic tree which fully supported *A. altissima* to be closely related with *Leitneria floridana* with 100% bootstrap value. The ML resolved 26 nodes with high branch support (over 98% bootstrap values), however six nodes were moderately supported perhaps as a result of less samples use (59 to 95%). Concerning relationships among families within Sapindales order, family Simaroubaceae early diverged and formed a sister clade/relationship with a 95% bootstrap support to Rutaceae family. Interestingly, the placement of families within Sapindales in our phylogenetic tree supports the one reported by previous studies [30,56,57] based on some chloroplast and nuclear markers. The families Anacardiaceae and Burseraceae formed a sister clade/ group, this clade further branched forming a sister clade with families Sapindaceae, Meliaceae, Simaroubaceae and Rutaceae analyzed in our study. Therefore, it is crucial to use more species for better understanding of Simaroubaceae phylogeny and evolution. This study provides a basis for future phylogenetic of Simaroubaceae species.

## 3. Materials and Methods

### 3.1. Plant Materials and DNA Extraction

Fresh leaves of *Ailanthus altissima* were collected in Wuhan Botanical Garden, Chinese Academy of Sciences in China. Total genomic DNA isolation was carried out using MagicMag Genomic DNA Micro Kit (Sangon Biotech Co., Shanghai, China) based on the manufactures protocol. The quality and integrity of DNA were checked and inspected using spectrophotometry and agarose gel electrophoresis respectively. The voucher specimen (HIB-LZZ-CC003) has been deposited at the Wuhan Botanical Garden herbarium (HIB) Wuhan, China.

### 3.2. The Tree of Heaven Plastome Sequence Assembly and Annotation

Library preparation was constructed using the Illumina Hiseq 2500 platform at NOVOgene Company (Beijing, China) with an average insert size of approximately 350 bp. The high-quality data (5 Gb) were filtered from raw sequence data (5.2 Gb) using the PRINSEQ lite v0.20.4 (San Diego State University, San Diego, CA, USA) [58] (phredQ ≥ 20, Length ≥ 50), followed by de novo assembling using NOVOPlasty [59] with default sets (K-mer = 31). The seeds and reference plastome used was from the closely related species *Leitneria floridana* (NC_030482) with high coverage of chloroplast reads ~1500×. Lastly, one contig of *Ailanthus altissima* was generated and mapped with reference plastome using GENEIOUS 8.1 (Biomatters Ltd., Auckland, New Zealand) [60]. Finally, online web-based server local blast was used to verify the inverted repeat (IR) single copy (SC) junctions.

Preliminary gene annotation of assembled genome was done using the program DOGMA (Dual Organellar GenoMe Annotator, University of Texas at Austin, Austin, TX, USA) [61], and BLAST (http://blast.ncbi.nlm.nih.gov/). The positions of start and stop codons together with position of introns were confirmed by comparing with homologous genes of other chloroplast genomes available at the GenBank database. Moreover, tRNA genes were verified with tRNAscan-SE server (http://lowelab.ucsc.edu/tRNAscan-SE/) [62]. The chloroplast genome physical circular map was drawn using program OGDRAW (Organellar Genome DRAW) [63] Max planck Institute of Molecular Plant Physiology, Potsdam, Germany) accompanied by manual corrections. The chloroplast genome sequence of *A. altissima* was deposited in the GenBank database, accession (MG799542). 

### 3.3. Genome Comparison and Gene Rearrangement

The border region between Inverted repeat (IR) and large single copy (LSC), also between inverted repeats and small single copy (SSC) junction were compared among seven representative species from Sapindales order. Additionally, alignments of seven chloroplast with one reference genome to determined gene rearrangements was carried out using Mauve v.4.0 [64].

### 3.4. Repeat Analysis in A. altissima Chloroplast Genome

Microsatellites were identified in the tree of heaven chloroplast genome and other selected representative genomes belonging to order Sapindales using an online software MIcroSAtellite (MISA) [65].The minimum number of repetitions were set to eight repeat units for mononucleotide SSR motifs, five repeat units for dinucleotide SSRs, four for trinucleotide SSRs and three repeat units for tetra-, penta-, and hexanucleotide motifs. The REPuter (https://bibiserv.cebitec.uni-bielefeld.de/reputer) program [66] (University of Bielefeld, Bielefeld, Germany) with default parameters was used to identify the location and sizes of forward, palindromic, complement and reverse repeat sequences in *A. altissima* chloroplast genome. 

### 3.5. Codon Usage and RNA Editing Sites

CodonW1.4.2 (http://downloads.fyxm.net/CodonW-76666.html) was used to analyze codon usage. Subsequently, possible RNA editing sites in *A. altissima* protein coding genes were predicted using the program predictive RNA editor for plants (PREP) suite [67] with the cutoff value set to 0.8. PREP server uses 35 genes as reference for potential RNA editing sites prediction by comparing the predicted protein genes to homologous proteins from other plants. 

### 3.6. Phylogenetic Analysis

Seventy five protein coding sequences present in 31 species from order Sapindales and three species from Thymelaeaceae (*Aquilaria sinensis*), Malvaceae (*Theobroma cacao* and *Abelmoschus esculentus*) as outgroups were used for the phylogenetic reconstruction. These species chloroplast genomes were downloaded from GenBank (Table S5). The protein coding sequences alignment was done using GENEIOUS v8.0.2 (Biomatters Ltd., Auckland, New Zealand) [60]. Maximum likelihood (ML) analysis was carried out using RAxMLversion 8.0.20 (Scientific Computing Group, Heidelberg Institute for Theoretical Studies, Institute of Theoretical Informatics, Karlsruhe Institute of Technology, Karlsruhe, Germany) [68] with 1000 replicates for bootstrap test. Lastly, the jModelTest v2.1.7 [69] was used to select the best substitution model (GTR + I + G). 

## 4. Conclusions

In this study, we present the plastome of tree of heaven from family Simaroubaceae which contains about 22 genera and over 100 species. *A. altissima* chloroplast genome possess circular and quadripartite structure which is well conserved similar to other plants chloroplast genomes. Nonetheless, the plastome showed slight variations at the four boundary junctions due to expansion and contraction in SC and IR borders. About 219 SSR loci and 49 repeats sequences were identified in *A. altissima* genome, this provides genetic information for designing DNA molecular markers for analyzing gene pool dynamics and genetic diversity of *A. altissima* natural populations aiming dispersal mechanism of this invasive tree. The phylogenetic analysis performed using 75 protein coding genes of 34 species available at the GenBank database, comprising 3 outgroup species from Malvales and 31 species representing families from order Sapindales. The two species from family Simaroubaceae formed a cluster and were group together with other families to form a single clade (Sapindales). In addition, the RNA editing analysis in *A. altissima* genome identified a total of 54 possible editing sites in 21 chloroplast genes with C-to-U transitions being the most. The availability of this chloroplast genome provides a tool to advance the study of evolution and invasion in *A. altissima* in order to address present evolutionary, ecological and genetic questions regarding this species.

## Figures and Tables

**Table 1:** List of genes found in *Ailanthus altissima* Chloroplast genome.

Functional Category	Group of Genes	Gene Name	Number
Self-replication	rRNA genes	*rrn16*(×2), *rrn23*(×2), *rrn4.5*(×2), *rrn5*(×2),	4
	tRNA genes	trnA-UGC*(×2), *trnC-GCA*, *trnD-GUC*, *trnE-UUC*, *trnF-GAA*, *trnG-UCC*, *trnH-GUG*, *trnI-CAU*(×2), trnI-GAU*(×2), trnK-UUU*, *trnL-CAA*(×2), trnL-UAA*, *trnL-UAG*, trnG-GCC*, *trnM-CAU*, *trnN-GUU*(×2), *trnP-GGG, trnP-UGG*, *trnQ-UUG*, *trnR-ACG*(×2), *trnR-UCU*, *trnS-GCU*, *trnS-GGA*, *trnS-UGA*, *trnT-GGU*, *trnT-UGU*, *trnV-GAC*(×2), trnV-UAC*, *trnW-CCA*, *trnY-GUA*	30
	Ribosomal small subunit	*rps2*, *rps3*, *rps4*, *rps7*(×2), *rps8*, *rps11*, *rps12*, *rps14*, *rps15*, rps16*, *rps18*, *rps19*	12
	Ribosomal large subunit	rpl2*(×2), *rpl14*, *rpl16*, *rpl20*, *rpl22*, *rpl23*(×2), *rpl32*, *rpl33*, *rpl36*	9
	DNA-dependent RNA polymerase	*rpoA*, *rpoB*, rpoC1*, *rpoC2*	4
Photosynthesis	Large subunit of rubisco	*rbcL*	1
	Photosystem I	*psaA*, *psaB*, *psaC*, *psaI*, *psaJ*, ycf3**	6
	Photosystem II	*psbA*, *psbB*, *psbC*, *psbD*, *psbE*, *psbF*, *psbH*, *psbI*, *psbJ*, *psbK*, *psbL*, *psbM*, *psbN*, *psbT*, *psbZ*	15
	NADH dehydrogenase	ndhA*, ndhB*(×2), *ndhC*, *ndhD*, *ndhE*, *ndhF*, *ndhG*, *ndhH*, *ndhI*, *ndhJ*, *ndhK*	11
	Cytochrome b/f complex	*petA*, petB*, *petD*, *petG*, *petL*, *petN*	6
	ATP synthase	*atpA*, *atpB*, *atpE*, atpF*, *atpH*, *atpI*	6
Other	Maturase	*matK*	1
	Subunit of acetyl-CoA carboxylase	*accD*	1
	Envelope membrane protein	*cemA*	1
	Protease	clpP**	1
	c-type cytochrome synthesis	*ccsA*	1
Functions unknown	Conserved open reading frames (*ycf*)	*ycf1*, *ycf2*(×2), *ycf4*, *ycf15*(×2)	4
Total			113

Note: * Gene with one intron, ** Genes with two introns. (×2) Genes with two copies.

**Table 2:** Predicted RNA editing site in the *A. altissima* chloroplast genome.

Gene	Nucleotide Position	Amino Acid Position	Codon Conversion	Amino Acid Conversion	Score
*accD*	818	273	T**C**G ≥ TTG	S ≥ L	0.80
*atpF*	92	31	C**C**A ≥ CTA	P ≥ L	0.86
	353	118	T**C**A ≥ TTA	S ≥ L	1.00
*atpB*	403	135	**C**CA ≥ TCA	P ≥ S	0.86
*rps14*	80	27	T**C**A ≥ TTA	S ≥ L	1.00
	149	50	T**C**A ≥ TTA	S ≥ L	1.00
*ccsA*	145	49	**C**TT ≥ TTT	L ≥ F	1.00
*clpP*	556	186	**C**AT ≥ TAT	H ≥ Y	1.00
*MatK*	319	107	**C**TT ≥ TTT	L ≥ F	0.86
	457	153	**C**AC ≥ TAC	H ≥ Y	1.00
	643	215	**C**AT ≥ TAT	H ≥ Y	1.00
	1246	416	**C**AC ≥ TAC	H ≥ Y	1.00
*ndhA*	107	36	C**C**T ≥ CTT	P ≥ L	1.00
	341	114	T**C**A ≥ TTA	S ≥ L	1.00
	566	189	T**C**A ≥ TTA	S ≥ L	1.00
	1073	358	T**C**C ≥ TTC	S ≥ F	1.00
*ndhB*	149	50	T**C**A ≥ TTA	S ≥ L	1.00
	467	156	C**C**A ≥ CTA	P ≥ L	1.00
	586	196	**C**AT ≥ TAT	H ≥ Y	1.00
	611	204	T**C**A ≥ TTA	S ≥ L	0.80
	746	249	T**C**T ≥ TTT	S ≥ F	1.00
	830	277	T**C**A ≥ TTA	S ≥ L	1.00
	836	279	T**C**A ≥ TTA	S ≥ L	1.00
	1255	419	**C**AT ≥ TAT	H ≥ Y	1.00
	1481	494	C**C**A ≥ CTA	P ≥ L	1.00
*ndhD*	2	1	A**C**G ≥ ATG	T ≥ M	1.00
	313	105	**C**GG ≥ TGG	R ≥ W	0.80
	383	128	T**C**A ≥ TTA	S ≥ L	1.00
	674	225	T**C**A ≥ TTA	S ≥ L	1.00
	878	293	T**C**A ≥ TTA	S ≥ L	1.00
	887	296	C**C**T ≥ CTT	P ≥ L	1.00
	1076	359	G**C**T ≥ GTT	A ≥ V	1.00
	1298	433	T**C**A ≥ TTA	S ≥ L	0.80
	1310	437	T**C**A ≥ TTA	S ≥ L	0.80
*ndhF*	290	97	T**C**A ≥ TTA	S ≥ L	1.00
	586	196	**C**TT ≥ TTT	L ≥ F	0.80
	1919	640	G**C**T ≥ GTT	A ≥ V	0.80
*ndhG*	166	56	**C**AT ≥ TAT	H ≥ Y	0.80
	320	107	A**C**A ≥ ATA	T ≥ I	0.80
*petL*	119	40	C**C**T ≥ CTT	P ≥ L	0.86
*psbF*	77	26	T**C**T ≥ TTT	S ≥ F	1.00
*rpl20*	308	103	T**C**A ≥ TTA	S ≥ L	0.86
*rpoA*	830	277	T**C**A ≥ TTA	S ≥ L	1.00
*rpoB*	338	113	T**C**T ≥ TTT	S ≥ F	1.00
	551	184	T**C**A ≥ TTA	S ≥ L	1.00
	566	189	T**C**G ≥ TTG	S ≥ L	1.00
	2426	809	T**C**A ≥ TTA	S ≥ L	0.86
*rpoC1*	41	14	T**C**A ≥ TTA	S ≥ L	1.00
*rpoC2*	1681	561	**C**AT ≥ TAT	H ≥ Y	0.86
	2030	677	A**C**T ≥ ATT	T ≥ I	1.00
	2314	772	**C**GG ≥ TGG	R ≥ W	1.00
	4183	1395	**C**TT ≥ TTT	L ≥ F	0.80
*rps2*	248	83	T**C**A ≥ TTA	S ≥ L	1.00
*rps16*	209	70	T**C**A ≥ TTA	S ≥ L	0.83

The cytidines marked are putatively edited to uredines.

**Table 3:** List of RNA editing sites shared by the seven plastomes predicted by PREP program.

Gene	A.A Position	*Citrus aurantiifolia*	*Rhus chinensis*	*Dodonaea viscosa*	*Boswellia Sacra*	*Leitneria floridana*	*Azadirachta indica*	*Ailanthus altissima*
		Codon (A.A) Conversion						
*atpF*	31	CCA (P) ≥ CTA (L)	CCA (P) ≥ CTA (L)	CCA (P) ≥ CTA (L)	CCA (P) ≥ CTA (L)	CCA (P) ≥ CTA (L)	CCA (P) ≥ CTA (L)	CCA (P) ≥ CTA (L)
*clpP*	187	CAT (H) ≥ TAT (Y)	CAT (H) ≥ TAT (Y)	CAT (H) ≥ TAT (Y)	CAT (H) ≥ TAT (Y)	CAT (H) ≥ TAT (Y)	CAT (H) ≥ TAT (Y)	CAT (H) ≥ TAT (Y)
*MatK*		CAT (H) ≥ TAT (Y)	CAT (H) ≥ TAT (Y)	CAT (H) ≥ TAT (Y)	CAT (H) ≥ TAT (Y)	CAT (H) ≥ TAT (Y)	CAT (H) ≥ TAT (Y)	CAT (H) ≥ TAT (Y)
*ndhA*	358	TCA (S) ≥ TTA (L)	TCA (S) ≥ TTA (L)	TCA (S) ≥ TTA (L)	TCA (S) ≥ TTA (L)	TCA (S) ≥ TTA (L)	TCA (S) ≥ TTA (L)	TCA (S) ≥ TTA (L)
*ndhB*	50	TCA (S) ≥ TTA (L)	TCA (S) ≥ TTA (L)	TCA (S) ≥ TTA (L)	TCA (S) ≥ TTA (L)	TCA (S) ≥ TTA (L)	TCA (S) ≥ TTA (L)	TCA (S) ≥ TTA (L)
	156	CCA (P) ≥ CTA (L)	CCA (P) ≥ CTA (L)	CCA (P) ≥ CTA (L)	CCA (P) ≥ CTA (L)	CCA (P) ≥ CTA (L)	CCA (P) ≥ CTA (L)	CCA (P) ≥ CTA (L)
	196	CAT (H) ≥ TAT (Y)	CAT (H) ≥ TAT (Y)	CAT (H) ≥ TAT (Y)	CAT (H) ≥ TAT (Y)	CAT (H) ≥ TAT (Y)	CAT (H) ≥ TAT (Y)	CAT (H) ≥ TAT (Y)
	249	TCT (S) ≥ TTT (F)	TCT (S) ≥ TTT (F)	TCT (S) ≥ TTT (F)	TCT (S) ≥ TTT (F)	TCT (S) ≥ TTT (F)	TCT (S) ≥ TTT (F)	TCT (S) ≥ TTT (F)
	419	CAT (H) ≥ TAT (Y)	CAT (H) ≥ TAT (Y)	CAT (H) ≥ TAT (Y)	CAT (H) ≥ TAT (Y)	CAT (H) ≥ TAT (Y)	CAT (H) ≥ TAT (Y)	CAT (H) ≥ TAT (Y)
*ndhD*	1	ACG (T) ≥ ATG (M)	ACG (T) ≥ ATG (M)	ACG (T) ≥ ATG (M)	ACG (T) ≥ ATG (M)	ACG (T) ≥ ATG (M)	ACG (T) ≥ ATG (M)	ACG (T) ≥ ATG (M)
	128	TCA (S) ≥ TTA (L)	TCA (S) ≥ TTA (L)	TCA (S) ≥ TTA (L)	TCA (S) ≥ TTA (L)	TCA (S) ≥ TTA (L)	TCA (S) ≥ TTA (L)	TCA (S) ≥ TTA (L)
*ndhG*	107	ACA (T) ≥ ATA (I)	ACA (T) ≥ ATA (I)	ACA (T) ≥ ATA (I)	ACA (T) ≥ ATA (I)	ACA (T) ≥ ATA (I)	ACA (T) ≥ ATA (I)	ACA (T) ≥ ATA (I)
*rpoA*	278	TCA (S) ≥ TTA (L)	TCA (S) ≥ TTA (L)	TCA (S) ≥ TTA (L)	TCA (S) ≥ TTA (L)	TCA (S) ≥ TTA (L)	TCA (S) ≥ TTA (L)	TCA (S) ≥ TTA (L)
*rpoB*	113	TCT (S) ≥ TTT (F)	TCT (S) ≥ TTT (F)	TCT (S) ≥ TTT (F)	TCT (S) ≥ TTT (F)	TCT (S) ≥ TTT (F)	TCT (S) ≥ TTT (F)	TCT (S) ≥ TTT (F)
	184	TCA (S) ≥ TTA (L)	TCA (S) ≥ TTA (L)	TCA (S) ≥ TTA (L)	TCA (S) ≥ TTA (L)	TCA (S) ≥ TTA (L)	TCA (S) ≥ TTA (L)	TCA (S) ≥ TTA (L)
	809	TCG (S) ≥ TTG (L)	TCG (S) ≥ TTG (L)	TCG (S) ≥ TTG (L)	TCG (S) ≥ TTG (L)	TCG (S) ≥ TTG (L)	TCG (S) ≥ TTG (L)	TCG (S) ≥ TTG (L)
*rpoC1*	14	TCA (S) ≥ TTA (L)	TCA (S) ≥ TTA (L)	TCA (S) ≥ TTA (L)	TCA (S) ≥ TTA (L)	TCA (S) ≥ TTA (L)	TCA (S) ≥ TTA (L)	TCA (S) ≥ TTA (L)
*rpoC2*	563	CAT (H) ≥ TAT (Y)	CAT (H) ≥ TAT (Y)	CAT (H) ≥ TAT (Y)	CAT (H) ≥ TAT (Y)	CAT (H) ≥ TAT (Y)	CAT (H) ≥ TAT (Y)	CAT (H) ≥ TAT (Y)
*rps14*	27	TCA (S) ≥ TTA (L)	TCA (S) ≥ TTA (L)	TCA (S) ≥ TTA (L)	TCA (S) ≥ TTA (L)	TCA (S) ≥ TTA (L)	TCA (S) ≥ TTA (L)	TCA (S) ≥ TTA (L)

**Table 4:** Distribution and localization of repetitive sequences F, forward: P, palindromic, R; reverse in *A. altissima* chloroplast genome.

Number	Size	Position 1	Type	Position 2	Location 1 (2)	Region
1	48	95,957	F	95,975	*ycf2*	IRa
2	48	153,174	F	153,192	*ycf2*	IRb
3	37	103,326	F	125,821	*rps12/trnV-GAC*(ndhA*)	IRa/SSC
4	30	95,957	F	95,993	*ycf2*	IRa
5	30	153,174	F	153,210	*ycf2*	IRb
6	29	50,944	F	50,972	trnL-UAA*	LSC
7	29	58,040	F	58,078	*rbcL*	LSC
8	28	115,434	F	115,460	*ycf1*	SSC
9	26	39,399	F	39,625	*psbZ/trnG-UCC*	LSC
10	25	71,153	F	71,178	*trnP-GGG/psaJ*	LSC
11	23	47,036	F	103,323	ycf3**(*rps12/trnV-GAC*)	LSC/IRa
12	23	112,456	F	112,488	*rrn4.5/rrn5*	IRa
13	23	136,686	F	136,718	*rrn5/rrn4.5*	IRb
14	22	11,749	F	11,771	*trnR-UCU/atpA*	LSC
15	21	248	F	270	*trnH-GUG/psbA*	LSC
16	21	9541	F	38,293	*trnS-GCU* (*trnS-UGA*)	LSC
17	21	41,956	F	44,180	*psaB(psaA)*	LSC
18	21	49,678	F	49,699	trnL-UAA*	LSC
19	20	1945	F	1965	*trnK-UUU*	LSC
20	20	15,166	F	92,503	*atpH/atpI*(*ycf2*)	LSC
21	20	47,039	F	125,821	ycf3**(*rps15*)	LSC/IRa
22	20	88,907	F	160,270	*rpl2*	IRa/IRb
25	48	31,790	P	31,790	*petN/psbM*	LSC
26	48	95,957	P	153,174	*ycf2*	IRa/IRb
27	48	95,975	P	153,192	*ycf2*	IRa/IRb
28	37	125,821	P	145,834	ndhA*(*trnV-GAC/rps12*)	SSC/IRb
29	36	30,970	P	30,970	*petN/psbM*	LSC
30	30	72,117	P	72,117	*rpl33/rps18*	LSC
31	30	95,957	P	153,174	*ycf2*	IRa/IRb
32	30	95,993	P	153,210	*ycf2*	IRa/IRb
33	27	542	P	571	*trnH-GUG/psbA*	LSC
34	25	11,403	P	11,430	*trnS-GCU/trnR-UCU*	LSC
35	24	4867	P	4897	*trnK-UUU/rps16*	LSC
36	24	9535	P	48,164	*trnS-GCU(psaA/ycf3)*	LSC
37	23	47,036	P	145,851	ycf3**(*trnV-GAC/rps12*)	LSC/IRb
38	23	51,804	P	119,066	*trnF-GAA/ndhJ*(*rpl32/trnL-UAG*)	LSC/SSC
39	23	112,456	P	136,686	*rrn4.5/rrn5*	IRa/IRb
40	23	112,488	P	136,718	*rrn4.5/rrn5*	IRa/IRb
41	22	39,195	P	39,195	*psbZ/trnG-UCC*	LSC
42	20	15,166	P	156,674	*atpH(ycf2)*	LSC/IRb
43	20	38,361	P	48,100	*trnS-UGA(trnS-GGA)*	LSC
44	20	88,907	P	88,907	*rpl2*	IRa
45	20	107,097	P	107,130	*rrn16/trnI-GAU*	IRa
46	23	39,184	R	39,184	*psbZ/trnG-UCC*	LSC
47	21	9751	R	9751	*trnS-GCU/trnR-UCU*	LSC
48	21	51,281	R	51,281	*trnL-UAA/trnF-GAA*	LSC
49	21	85,055	R	85,055	*rps8/rpl14*	LSC
50	20	53,712	R	53,712	*ndhC*	LSC
51	20	9385	R	13,356	*psbI*(*atpA/atpF*)	LSC

F: forward; P: palindrome; R; reverse* intron or ** introns.

## Supplementary Materials

Supplementary materials can be found at http://www.mdpi.com/1422-0067/19/4/929/s1.

## Abbreviations

SC	Single copy
LSC	Large single copy
SSC	Small single copy
IR	Inverted repeat
