# Supplementary material for: The Complete Chloroplast Genome Sequence of Tree of Heaven (Ailanthus altissima (Mill.) (Sapindales: Simaroubaceae), an Important Pantropical Tree
Source: Int J Mol Sci. 2018 Mar 21;19(4):929. doi: 10.3390/ijms19040929 (PMC5979363; doi:10.3390/ijms19040929)
Supplement: Supplementary file 1 [file ijms-19-00929-s001.pdf]

Supplementary Materials:

Table S1. Codon usage in all the protein coding genes.

Amino acid	codon	Number	RSCU	Proportion (%)	Amino acid	codon	Number	RSCU	Proportion (%)
Phe	UUU	861	1.31	5.71	Ser	UCU	452	1.59	7.73
	UUC	450	0.69			UCC	274	0.96	
Leu	UUA	764	1.89	10.56		UCA	340	1.2	
	UUG	502	1.24			UCG	173	0.61	
	CUU	499	1.23			AGU	391	1.72	
	CUC	154	0.38			AGC	144	0.63	
	CUA	338	0.84		Pro	CCU	359	1.51	4.15
	CUG	168	0.42			CCC	189	0.79	
Ile	AUU	974	1.49	8.54		CCA	267	1.12	
	AUC	394	0.6			CCG	138	0.58	
	AUA	593	0.91		Thr	ACU	451	1.55	5.07
Met	AUG	536	1	2.33		ACC	230	0.79	
Val	GUU	476	1.49	5.58		ACA	350	1.2	
	GUC	156	0.49			ACG	133	0.46	
	GUA	489	1.53		Ala	GCU	571	1.77	5.63
	GUG	160	0.5			GCC	203	0.63	
Tyr	UAU	684	1.64	3.64		GCA	351	1.09	
	UAC	152	0.36			GCG	169	0.52	
His	CAU	401	1.45	2.41	Cys	UGU	188	1.45	1.13
	CAC	152	0.55			UGC	71	0.55	
Gln	CAA	626	1.55	3.51	Trp	UGG	416	1	1.81
	CAG	181	0.45		Arg	AGA	282	1.24	5.64
Asn	AAU	848	1.54	4.79		AGG	108	0.47	
	AAC	251	0.46			CGU	330	1.45	
Lys	AAA	897	1.51	5.19		CGC	110	0.48	
	AAG	294	0.49			CGA	360	1.27	
Asp	GAU	719	1.58	3.97		CGG	105	0.37	
	GAC	193	0.42		Gly	GGU	524	1.31	6.99
Glu	GAA	922	1.52	5.28		GGC	174	0.43	
	GAG	290	0.48			GGA	628	1.56	
						GGG	280	0.7	

Table S2. Codon usage for individual protein genes

Gene	T3s	C3s	A3s	G3s	CAI	CBI	Fop	Nc	GC3s	GC	L_sym	L_aa	Gravy	Aromo
psbA	0.52	0.26	0.29	0.10	0.30	0.20	0.54	41.68	0.31	0.43	331.00	353.00	0.35	0.14
matK	0.48	0.19	0.44	0.18	0.16	-0.14	0.32	50.38	0.28	0.34	488.00	505.00	-0.12	0.14
atpA	0.44	0.19	0.43	0.16	0.19	-0.02	0.41	48.51	0.28	0.41	499.00	510.00	-0.06	0.06
atpF	0.42	0.15	0.47	0.25	0.15	-0.16	0.32	41.92	0.30	0.38	202.00	209.00	-0.23	0.09
atpI	0.49	0.19	0.39	0.11	0.18	-0.05	0.37	45.81	0.25	0.38	238.00	247.00	0.70	0.13
rps2	0.47	0.14	0.43	0.20	0.18	-0.16	0.33	48.83	0.26	0.39	225.00	236.00	-0.27	0.07
rpoC2	0.45	0.17	0.44	0.20	0.15	-0.14	0.33	51.58	0.29	0.38	1362.00	1401.00	-0.27	0.09
rpoC1	0.48	0.15	0.45	0.18	0.15	-0.12	0.34	49.01	0.25	0.39	662.00	684.00	-0.28	0.10
rpoB	0.45	0.13	0.45	0.23	0.15	-0.12	0.34	49.72	0.27	0.39	1037.00	1070.00	-0.25	0.09
psbD	0.51	0.21	0.36	0.13	0.25	0.05	0.45	46.15	0.28	0.43	331.00	353.00	0.36	0.17
psbC	0.47	0.20	0.38	0.14	0.20	0.01	0.42	46.20	0.28	0.44	448.00	473.00	0.26	0.14
rps14	0.40	0.10	0.47	0.27	0.14	-0.13	0.33	**38.94**	0.29	0.42	96.00	100.00	-1.01	0.06
psaB	0.50	0.17	0.36	0.18	0.19	-0.08	0.37	48.79	0.28	0.42	689.00	734.00	0.13	0.15
psaA	0.45	0.19	0.39	0.18	0.19	-0.10	0.36	50.11	0.30	0.43	707.00	750.00	0.25	0.13
ycf3	0.45	0.19	0.42	0.26	0.16	-0.15	0.35	57.78	0.32	0.41	162.00	169.00	-0.45	0.14
rps4	0.44	0.19	0.48	0.13	0.15	-0.03	0.38	52.37	0.25	0.39	197.00	201.00	-0.60	0.07
ndhJ	0.50	0.14	0.40	0.23	0.18	-0.12	0.34	53.19	0.28	0.40	149.00	158.00	-0.27	0.15
ndhK	0.47	0.17	0.42	0.17	0.18	-0.13	0.34	52.34	0.27	0.39	219.00	227.00	-0.19	0.10
ndhC	0.49	0.12	0.42	0.18	0.19	-0.10	0.34	52.80	0.23	0.36	110.00	120.00	1.02	0.21
atpE	0.44	0.18	0.41	0.20	0.16	-0.08	0.36	53.67	0.29	0.40	128.00	133.00	-0.05	0.02
atpB	0.44	0.19	0.39	0.18	0.21	0.02	0.42	48.79	0.30	0.44	484.00	498.00	-0.03	0.06
rbcL	0.47	0.21	0.39	0.16	0.24	0.04	0.44	51.95	0.30	0.45	458.00	475.00	-0.26	0.10
accD	0.57	0.17	0.36	0.20	0.19	-0.18	0.34	49.04	0.27	0.35	474.00	496.00	-0.49	0.11
ycf4	0.45	0.19	0.40	0.18	0.15	-0.11	0.35	51.15	0.29	0.39	175.00	184.00	0.19	0.17
cemA	0.50	0.21	0.42	0.17	0.19	-0.07	0.37	58.19	0.27	0.33	219.00	232.00	0.21	0.15
petA	0.47	0.17	0.36	0.25	0.18	-0.05	0.38	51.78	0.32	0.41	315.00	320.00	-0.10	0.08
rpl20	0.46	0.10	0.44	0.23	0.10	-0.20	0.29	47.39	0.24	0.36	113.00	117.00	-0.53	0.09
rps12	0.36	0.22	0.41	0.18	0.15	-0.05	0.37	51.70	0.33	0.46	117.00	118.00	-0.46	0.03
clpP	0.41	0.19	0.44	0.18	0.16	-0.11	0.33	**58.37**	0.30	0.43	183.00	195.00	0.17	0.09
psbB	0.49	0.17	0.38	0.15	0.19	-0.05	0.39	47.14	0.26	0.44	481.00	508.00	0.12	0.15
petB	0.45	0.18	0.37	0.20	0.21	-0.04	0.38	49.32	0.30	0.42	218.00	234.00	0.49	0.15
petD	0.43	0.15	0.44	0.15	0.16	-0.13	0.30	44.60	0.25	0.40	167.00	174.00	0.51	0.10
rpoA	0.47	0.16	0.46	0.21	0.18	-0.11	0.35	51.07	0.27	0.36	319.00	327.00	-0.29	0.07
rps11	0.42	0.13	0.47	0.10	0.15	-0.16	0.32	46.85	0.20	0.46	133.00	138.00	-0.37	0.04
rps8	0.41	0.16	0.48	0.16	0.10	-0.05	0.37	42.20	0.25	0.36	128.00	134.00	-0.25	0.06
rpl14	0.44	0.17	0.48	0.12	0.17	-0.06	0.37	47.58	0.23	0.39	119.00	122.00	-0.02	0.03
rpl16	0.38	0.14	0.55	0.10	0.12	-0.09	0.36	37.99	0.20	0.42	127.00	136.00	-0.48	0.08
rps3	0.41	0.18	0.59	0.08	0.15	-0.13	0.34	44.62	0.20	0.36	212.00	219.00	-0.34	0.07
rpl22	0.42	0.18	0.48	0.19	0.15	-0.14	0.34	56.04	0.28	0.37	155.00	161.00	-0.43	0.09
rpl2	0.39	0.20	0.43	0.19	0.14	-0.10	0.36	54.14	0.31	0.44	269.00	275.00	-0.56	0.05
ycf2	0.44	0.21	0.40	0.26	0.16	-0.14	0.34	53.61	0.35	0.38	2208.00	2299.00	-0.43	0.12
ndhB	0.43	0.21	0.42	0.14	0.17	-0.06	0.37	48.57	0.28	0.38	483.00	510.00	0.67	0.13
rps7	0.42	0.15	0.52	0.12	0.19	-0.04	0.39	44.23	0.21	0.41	150.00	155.00	-0.60	0.05
ndhF	0.51	0.14	0.43	0.16	0.14	-0.19	0.30	46.33	0.23	0.33	706.00	746.00	0.59	0.17
ccsA	0.47	0.15	0.46	0.15	0.13	-0.22	0.28	48.89	0.23	0.33	300.00	319.00	0.54	0.15
ndhD	0.41	0.16	0.43	0.20	0.13	-0.15	0.31	51.55	0.28	0.37	478.00	500.00	0.77	0.15

ndhE	0.51	0.16	0.36	0.18	0.18	-0.08	0.36	47.77	0.26	0.35	97.00	101.00	0.69	0.10
ndhG	0.49	0.12	0.38	0.20	0.14	-0.20	0.27	47.50	0.24	0.35	172.00	178.00	1.09	0.12
ndhI	0.55	0.15	0.45	0.13	0.22	-0.11	0.35	43.21	0.21	0.35	164.00	170.00	-0.16	0.11
ndhA	0.45	0.14	0.46	0.12	0.13	-0.13	0.31	41.83	0.21	0.36	350.00	363.00	0.69	0.12
ndhH	0.48	0.15	0.48	0.16	0.17	-0.05	0.38	49.57	0.23	0.39	367.00	393.00	-0.10	0.11
ycf1	0.48	0.17	0.53	0.17	0.17	-0.11	0.36	49.03	0.24	0.32	1807.00	1886.00	-0.62	0.12

Table S3. List of RNA editing sites predicted by PREP program in selected Sapindales Cp genomes

		Citrus aurantiifolia	*Rhus chinensis*	*Dodonaea viscosa*	*Boswellia Sacra*	*Leitneria floridana*	*Azadirachta indica*	*Ailanthus altissima*
Gene	A.A position	Codon (A.A) Conversion						
accD	472	CCT (P) => CTT (L)	TCG (S) => TTG (L)	**CTT (L) => TTT (F)**	**CTT (L) => TTT (F)**	TCG (S) => TTG (L)	TCG (S) => TTG (L)	TCG(S)=> TTG (L)
			CCT (P) => CTT (L)	TCT (S) => TTT (F)	TCG (S) => TTG (L)		CCT (P) => CTT (L)	
				TCG (S) => TTG (L)	CCT (P) => CTT (L)			
				**CCA (P) => TCA (S)**				
				CCT (P) => CTT (L)				
atpF	31	CCA (P) => CTA (L)	TCG (S) => TTG (L)	TCG (S) => TTG (L)	CCA (P) => CTA (L)	CCA (P) => CTA (L)	CCA (P) => CTA (L)	CCA (P) => CTA (L)
			CCA (P) => CTA (L)	CCA (P) => CTA (L)				TCA (S) => TTA (L)
clpP	187	**CAT (H) => TAT (Y)**	**CAT (H) => TAT (Y)**	**CTT (L) => TTT (F)**	**CTT (L) => TTT (F)**	**CAT (H) => TAT (Y)**	**CAT (H) => TAT (Y)**	**CAT (H) => TAT (Y)**
				**CAT (H) => TAT (Y)**	**CAT (H) => TAT (Y)**			
MatK	215	**CAT (H) => TAT (Y)**	**CAC (H) => TAC (Y)**	**CAT (H) => TAT (Y)**	**CAC (H) => TAC (Y)**	**CAT (H) => TAT (Y)**	TCA (S) => TTA (L)	**CTT (L) => TTT (F)**
			**CAT (H) => TAT (Y)**	**CAC (H) => TAC (Y)**	**CAT (H) => TAT (Y)**	**CAC (H) => TAC (Y)**	**CAC (H) => TAC (Y)**	**CAC (H) => TAC (Y)**
			**CGG (R) => TGG (W)**		**CGG (R) => TGG (W)**		TCC (S) => TTC (F)	**CAT (H) => TAT (Y)**
			**CAC (H) => TAC (Y)**		**CAC (H) => TAC (Y)**		**CAT (H) => TAT (Y)**	**CAC (H) => TAC (Y)**
							**CAT (H) => TAT (Y)**	
ndhA	114	TCA (S) => TTA (L)	CCT (P) => CTT (L)	**CAC (H) => TAC (Y)**	CCT (P) => CTT (L)	TCA (S) => TTA (L)	CCT (P) => CTT (L)	CCT (P) => CTT (L)
	358	TCC (S) => TTC (F)	TCA (S) => TTA (L)	**CCT (P) => TCT (S)**	TCA (S) => TTA (L)	TCA (S) => TTA (L)	TCA (S) => TTA (L)	TCA (S) => TTA (L)
			TCA (S) => TTA (L)	TCA (S) => TTA (L)	TCA (S) => TTA (L)	TCC (S) => TTC (F)	TCC (S) => TTC (F)	TCA (S) => TTA (L)
			TCC (S) => TTC (F)	TCC (S) => TTC (F)	TCC (S) => TTC (F)			TCC (S) => TTC (F)
ndhB	50	TCA (S) => TTA (L)	TCA (S) => TTA (L)	**CTC (L) => TTC (F)**	TCA (S) => TTA (L)	TCA (S) => TTA (L)	TCA (S) => TTA (L)	TCA (S) => TTA (L)
	156	CCA (P) => CTA (L)	CCA (P) => CTA (L)	TCA (S) => TTA (L)	CCA (P) => CTA (L)	CCA (P) => CTA (L)	CCA (P) => CTA (L)	CCA (P) => CTA (L)
	196	**CAT (H) => TAT (Y)**	**CAT (H) => TAT (Y)**	CCA (P) => CTA (L)	**CAT (H) => TAT (Y)**	**CAT (H) => TAT (Y)**	**CAT (H) => TAT (Y)**	**CAT (H) => TAT (Y)**
	204	TCA (S) => TTA (L)	TCA (S) => TTA (L)	**CAT (H) => TAT (Y)**	TCA (S) => TTA (L)	TCA (S) => TTA (L)	TCA (S) => TTA (L)	TCA (S) => TTA (L)
	249	TCT (S) => TTT (F)	CCA (P) => CTA (L)	TCA (S) => TTA (L)	TCT (S) => TTT (F)	TCT (S) => TTT (F)	CCA (P) => CTA (L)	TCT (S) => TTT (F)
	277	TCA (S) => TTA (L)	TCT (S) => TTT (F)	TCT (S) => TTT (F)	TCA (S) => TTA (L)	TCA (S) => TTA (L)	TCT (S) => TTT (F)	TCA (S) => TTA (L)
	279	TCA (S) => TTA (L)	TCA (S) => TTA (L)	TCA (S) => TTA (L)	TCA (S) => TTA (L)	TCA (S) => TTA (L)	TCA (S) => TTA (L)	TCA (S) => TTA (L)
	419	**CAT (H) => TAT (Y)**	TCA (S) => TTA (L)	TCA (S) => TTA (L)	TCA (S) => TTA (L)	**CAT (H) => TAT (Y)**	TCA (S) => TTA (L)	**CAT (H) => TAT (Y)**
			TCA (S) => TTA (L)	TCA (S) => TTA (L)	**CAT (H) => TAT (Y)**	CCA (P) => CTA (L)	**CAT (H) => TAT (Y)**	CCA (P) => CTA (L)
			**CAT (H) => TAT (Y)**	**CAT (H) => TAT (Y)**	CCA (P) => CTA (L)		CCA (P) => CTA (L)	
			CCA (P) => CTA (L)	**CTC (L) => TTC (F)**				
				CCA (P) => CTA (L)				
ndhD	1	ACG (T) => ATG (M)	ACG (T) => ATG (M)	ACG (T) => ATG (M)	ACG (T) => ATG (M)	ACG (T) => ATG (M)	ACG (T) => ATG (M)	ACG (T) => ATG (M)
	105	**CGG (R) => TGG (W)**	**CGG (R) => TGG (W)**	TCA (S) => TTA (L)	**CGG (R) => TGG (W)**	**CGG (R) => TGG (W)**	**CGG (R) => TGG (W)**	**CGG (R) => TGG (W)**
	128	TCA (S) => TTA (L)	TCA (S) => TTA (L)	TCA (S) => TTA (L)	TCA (S) => TTA (L)	TCA (S) => TTA (L)	TCA (S) => TTA (L)	TCA (S) => TTA (L)
	293	TCA (S) => TTA (L)	TCA (S) => TTA (L)	TCA (S) => TTA (L)	TCA (S) => TTA (L)	TCA (S) => TTA (L)	TCA (S) => TTA (L)	TCA (S) => TTA (L)
	296	CCC (P) => CTC (L)	TCA (S) => TTA (L)	CCT (P) => CTT (L)	TCA (S) => TTA (L)	TCA (S) => TTA (L)	CCT (P) => CTT (L)	TCA (S) => TTA (L)
	359	GCT (A) => GTT (V)	CCT (P) => CTT (L)	TCA (S) => TTA (L)	CCT (P) => CTT (L)	GCT (A) => GTT (V)	GCT (A) => GTT (V)	CCT (P) => CTT (L)
	433	TCA (S) => TTA (L)	TCA (S) => TTA (L)	TCA (S) => TTA (L)	TCA (S) => TTA (L)	TCA (S) => TTA (L)	TCA (S) => TTA (L)	GCT (A) => GTT (V)
	469	**CTT (L) => TTT (F)**	TCA (S) => TTA (L)	**CTT (L) => TTT (F)**	TCA (S) => TTA (L)	TCA (S) => TTA (L)	TCA (S) => TTA (L)	TCA (S) => TTA (L)
	487	GCG (A) => GTG (V)	**CTT (L) => TTT (F)**		**CTT (L) => TTT (F)**		**CTT (L) => TTT (F)**	TCA (S) => TTA (L)
	490	GCC (A) => GTC (V)						
ndhG	56	**CAT (H) => TAT (Y)**	**CAT (H) => TAT (Y)**	TCT (S) => TTT (F)	**CAT (H) => TAT (Y)**	CAT (H) => TAT (Y)	**CAT (H) => TAT (Y)**	**CAT (H) => TAT (Y)**
	107	ACA (T) => ATA (I)	ACA (T) => ATA (I)	ACA (T) => ATA (I)	ACA (T) => ATA (I)	ACA (T) => ATA (I)	ACA (T) => ATA (I)	ACA (T) => ATA (I)
ndhF	97	TCA (S) => TTA (L)	**CAT (H) => TAT (Y)**	**CAC (H) => TAC (Y)**	TCA (S) => TTA (L)	TCA (S) => TTA (L)	**CTT (L) => TTT (F)**	TCA (S) => TTA (L)
	196	**CTT (L) => TTT (F)**	TCA (S) => TTA (L)	TCA (S) => TTA (L)	TCG (S) => TTG (L)	**CTT (L) => TTT (F)**		**CTT (L) => TTT (F)**
	433	ACA (T) => ATA (I)	TCC (S) => TTC (F)	**CTT (L) => TTT (F)**	GCT (A) => GTT (V)	GCT (A) => GTT (V)		GCT (A) => GTT (V)
	576	TCA (S) => TTA (L)	**CTT (L) => TTT (F)**					
	619	**CTT (L) => TTT (F)**	GCT (A) => GTT (V)					
	642	GCT (A) => GTT (V)	**CTC (L) => TTC (F)**					
rpl20	103	TCA (S) => TTA (L)	GCT (A) => GTT (V)	**CAT (H) => TAT (Y)**	GCT (A) => GTT (V)	TCA (S) => TTA (L)	TCA (S) => TTA (L)	TCA (S) => TTA (L)
			TCA (S) => TTA (L)	TCA (S) => TTA (L)				
rpoA	278	TCA (S) => TTA (L)	GCT (A) => GTT (V)	**CGT (R) => TGT (C)**	TCA (S) => TTA (L)	TCA (S) => TTA (L)	TCA (S) => TTA (L)	TCA (S) => TTA (L)
			TCA (S) => TTA (L)	TCA (S) => TTA (L)				
rpoB	113	TCT (S) => TTT (F)	TCT (S) => TTT (F)	TCT (S) => TTT (F)	TCT (S) => TTT (F)	TCT (S) => TTT (F)	TCT (S) => TTT (F)	TCT (S) => TTT (F)
	184	TCA (S) => TTA (L)	TCA (S) => TTA (L)	TCA (S) => TTA (L)	TCA (S) => TTA (L)	TCA (S) => TTA (L)	TCA (S) => TTA (L)	TCA (S) => TTA (L)
	189	TCG (S) => TTG (L)	TCG (S) => TTG (L)	TCG (S) => TTG (L)	TCG (S) => TTG (L)	TCG (S) => TTG (L)	TCG (S) => TTG (L)	TCG (S) => TTG (L)
	809	TCA (S) => TTA (L)	TCA (S) => TTA (L)	TCA (S) => TTA (L)	TCA (S) => TTA (L)	TCA (S) => TTA (L)		TCA (S) => TTA (L)
				**CTT (L) => TTT (F)**				
				TCC (S) => TTC (F)				
rpoC1	14	TCA (S) => TTA (L)	TCA (S) => TTA (L)	TCA (S) => TTA (L)	TCA (S) => TTA (L)	TCA (S) => TTA (L)	TCA (S) => TTA (L)	TCA (S) => TTA (L)
				**CTT (L) => TTT (F)**				
rpoC2	280	**CTT (L) => TTT (F)**	**CAT (H) => TAT (Y)**	**CAT (H) => TAT (Y)**	ACA (T) => ATA (I)	**CAT (H) => TAT (Y)**	**CAT (H) => TAT (Y)**	**CAT (H) => TAT (Y)**
	484	**CGC (R) => TGC (C)**	ACT (T) => ATT (I)	ACT (T) => ATT (I)	**CAT (H) => TAT (Y)**	ACT (T) => ATT (I)	ACT (T) => ATT (I)	ACT (T) => ATT (I)
	563	**CAT (H) => TAT (Y)**	**CGG (R) => TGG (W)**	GCC (A) => GTC (V)	ACT (T) => ATT (I)	CGG (R) => TGG (W)	**CGG (R) => TGG (W)**	**CGG (R) => TGG (W)**
	670	**CTT (L) => TTT (F)**	**CTT (L) => TTT (F)**	**CCA (P) => TCA (S)**	**CGG (R) => TGG (W)**	CTT (L) => TTT (F)	**CTT (L) => TTT (F)**	**CTT (L) => TTT (F)**
	774	**CGG (R) => TGG (W)**			**CTT (L) => TTT (F)**		**CTT (L) => TTT (F)**	
rps14	27	TCA (S) => TTA (L)	TCA (S) => TTA (L)	TCA (S) => TTA (L)	TCA (S) => TTA (L)	TCA (S) => TTA (L)	TCA (S) => TTA (L)	TCA (S) => TTA (L)
	50	TCA (S) => TTA (L)	CCA (P) => CTA (L)	CCA (P) => CTA (L)	TCA (S) => TTA (L)	TCA (S) => TTA (L)	TCA (S) => TTA (L)	TCA (S) => TTA (L)

rps16	71	TCA (S) => TTA (L)	TCA (S) => TTA (L)		TCA (S) => TTA (L)	TCA (S) => TTA (L)	TCA (S) => TTA (L)	TCA (S) => TTA (L)
psbF	26	TCT (S) => TTT (F)	TCT (S) => TTT (F)		TCT (S) => TTT (F)	TCT (S) => TTT (F)	**CTT (L) => TTT (F)**	TCT (S) => TTT (F)
atpA	264	CCC (P) => CTC (L)	CCC (P) => CTC (L)		CCC (P) => CTC (L)	CCC (P) => CTC (L)	CCC (P) => CTC (L)	
ccsA	46	ACA (T) => ATA (I)		**CTT (L) => TTT (F)**	CTT (L) => TTT (F)			**CTT (L) => TTT (F)**
				**CTT (L) => TTT (F)**				
rps2			TCA (S) => TTA (L)		TCA (S) => TTA (L)	TCA (S) => TTA (L)	TCA (S) => TTA (L)	TCA (S) => TTA (L)
rps8	48	GCA (A) => GTA (V)						
rpl2	198	GCG (A) => GTG (V)						
psaI			TCT (S) => TTT (F)					
psaB				GCT (A) => GTT (V)				
atpB						**CCC (P) => TCC (S)**	**CCA (P) => TCA (S)**	**CCA (P) => TCA (S)**
rpl23							ACG (T) => ATG (M)	
petL								CCT (P) => CTT (L)
Total	Sites	52	61	61	57	48	53	54
	First codon	14	16	23	15	9	16	15
	Second codon	38	45	38	42	39	37	39

Table S4. List of RNA editing sites shared by the two species of Simaroubaceae family.

Gene	A.A position	Leitneria floridana<br>Codon (A.A) Conversion	Ailanthus altissima
accD	472	TCG (S) => TTG (L)	TCG(S)=> TTG (L)
clpP	187	CAT (H) => TAT (Y)	CAT (H) => TAT (Y)
MatK	215	CAT (H) => TAT (Y)	CAT (H) => TAT (Y)
		CAC (H) => TAC (Y)	CAC (H) => TAC (Y)
ndhA	358	TCA (S) => TTA (L)	TCA (S) => TTA (L)
		TCC (S) => TTC (F)	TCC (S) => TTC (F)
ndhB	50	TCA (S) => TTA (L)	TCA (S) => TTA (L)
	156	CCA (P) => CTA (L)	CCA (P) => CTA (L)
	196	CAT (H) => TAT (Y)	CAT (H) => TAT (Y)
	249	TCT (S) => TTT (F)	TCT (S) => TTT (F)
	419	CAT (H) => TAT (Y)	CAT (H) => TAT (Y)
		CCA (P) => CTA (L)	CCA (P) => CTA (L)
ndhD	1	ACG (T) => ATG (M)	ACG (T) => ATG (M)
	105	CGG (R) => TGG (W)	CGG (R) => TGG (W)
	128	TCA (S) => TTA (L)	TCA (S) => TTA (L)
	433	GCT (A) => GTT (V)	GCT (A) => GTT (V)
ndhG	56	CAT (H) => TAT (Y)	CAT (H) => TAT (Y)
	107	ACA (T) => ATA (I)	ACA (T) => ATA (I)
ndhF	97	TCA (S) => TTA (L)	TCA (S) => TTA (L)
	196	**CTT (L) => TTT (F)**	**CTT (L) => TTT (F)**
rpl20	103	TCA (S) => TTA (L)	TCA (S) => TTA (L)
rpoA	278	TCA (S) => TTA (L)	TCA (S) => TTA (L)
rpoB	113	TCT (S) => TTT (F)	TCT (S) => TTT (F)
	184	TCA (S) => TTA (L)	TCA (S) => TTA (L)
	809	TCG (S) => TTG (L)	TCG (S) => TTG (L)
rpoC1	14	TCA (S) => TTA (L)	TCA (S) => TTA (L)
rpoC2	280	CTT (L) => TTT (F)	CTT (L) => TTT (F)
	484	CGG (R) => TGG (W)	CGG (R) => TGG (W)
	563	CAT (H) => TAT (Y)	CAT (H) => TAT (Y)
	670	ACT (T) => ATT (I)	ACT (T) => ATT (I)
rps14	27	TCA (S) => TTA (L)	TCA (S) => TTA (L)
psbF	26	TCT (S) => TTT (F)	TCT (S) => TTT (F)
rps2		TCA (S) => TTA (L)	TCA (S) => TTA (L)

Table S5. List of species use for phylogenetic tree

	Species	GenBank Number	Family
1	*Mangifera indica*	NC_035239	Anacardiaceae
2	*Spondias-bahiensis*	NC_030526	
3	*Spondias-mombin*	NC_035973	
4	*Spondias-tuberosa*	NC_030527	
5	*Boswellia sacra*	NC_029420	Burseraceae
6	*Azadirachta indica*	NC_023792	Meliaceae
7	*Citrus aurantiifolia*	NC_024929	Rutaceae
8	*Citrus platymamma*	NC_030194	
9	*Clausena excavata*	NC_032685	
10	*Glycosmis mauritiana*	NC_032686	
11	*Glycosmis pentaphylla*	NC_032687	
12	*Merrillia caloxylon*	NC_032688	
13	*Micromelum minutum*	NC_032689	
14	*Murraya koenigii*	NC_032684	
15	*Phellodendron amurense*	NC_035551	
16	*Zanthoxylum piperitum*	NC_027939	
17	*Zanthoxylum schinifolium*	NC_030702	
18	*Acer buergerianum*	NC_034744	Sapindaceae
19	*Acer davidii*	NC_030331	
20	*Acer griseum*	NC_034346	
21	*Acer miaotaiense*	NC_030343	
22	*Acer morrisonense*	NC_029371	
23	*Acer palmatum*	NC_034932	
24	*Aesculus wangii*	NC_035955	
25	*Dipteronia dyeriana*	NC_031899	
26	*Dipteronia sinensis*	NC_029338	
27	*Dodonaea viscosa*	NC_036099	
28	*Litchi chinensis*	NC_035238	
29	*Sapindus mukorossi*	NC_025554	
30	*Leitneria floridana*	NC_030482	Simaroubaceae
31	*Ailanthus altissima*	MG_799542	
32	*Aquilaria sinensis*	NC_029243	Outgroup
33	*Abelmoschus esculentus*	NC_035234	
34	*Theobroma cacao*	NC_014676	
